# ZenGuard a machine learning based zero trust framework for context aware threat mitigation using SIEM SOAR and UEBA

**DOI:** 10.1038/s41598-025-20998-4

**Published:** 2025-10-14

**Authors:** Aamina Hassan, Abdul Rauf, Narmeen Shafqat, Rabia Latif, Hasib Khan

**Affiliations:** 1 National University of Sciences and Technology, Islamabad, Pakistan; 2 College of Computer and Information Sciences (CCIS), Prince Sultan University, Riyadh, 11586 Saudi Arabia; 3 Department of Mathematics and Sciences, Prince Sultan University, Riyadh, 11586 Saudi Arabia

**Keywords:** Zero Trust Architecture, SIEM, UEBA, SOAR, MFA, Threat Detection, Incident Response, Python Automation, Electrical and electronic engineering, Energy harvesting

## Abstract

Perimeter-based security models, which rely on predefined network boundaries, are increasingly ineffective against modern threats such as insider misuse, supply chain attacks, and Advanced Persistent Threats (APTs). Zero Trust Architecture (ZTA) offers a more resilient approach by enforcing continuous verification of users, devices, and activity. While SIEM (Security Information and Event Management) and SOAR (Security Orchestration, Automation, and Response) platforms are widely adopted and play a critical role in monitoring and response, they often operate with static rules and limited behavioral context, making it challenging to fully implement ZTA principles. ZenGuard addresses these operational gaps by introducing context-aware, real-time, and adaptive enforcement capabilities. This paper introduces ZenGuard, an open-source framework that integrates ZTA, SIEM, SOAR, and User and Entity Behavior Analytics (UEBA) into a unified, vendor-independent platform. ZenGuard employs Python-based automation and interpretable machine learning models to detect behavioral anomalies and trigger adaptive responses across identity, device, and network layers. We evaluate ZenGuard using real-world Security Operation Center (SOC) telemetry from enterprise environments to validate overall threat detection and response, demonstrating a Mean Time to Respond (MTTR) under 10 seconds in cases such as privilege escalation, lateral movement and data exfiltration. Furthermore, UEBA accuracy was assessed on synthetic behavioral datasets that emulate diverse threats that are not consistently observable in live environments. In essence, ZenGuard supports Zero Trust principles as defined by NIST SP 800-207 and ISO/IEC 27001 controls, offering a practical, explainable, and scalable approach to modern cybersecurity automation.

## Introduction

Organizations today face an increasingly complex threat landscape. Employees work remotely, devices connect from untrusted networks, and systems span hybrid cloud, third-party vendors, and on-premise environments. In such dynamic settings, attackers have more opportunities than ever to infiltrate systems through phishing, supply chain compromise, or malicious insiders—and move undetected across networks.

Historically, enterprises relied on *perimeter-based security models*, where everything inside the network was trusted and threats were expected to come from the outside. Firewalls, Virtual Private Networks (VPNs), and access controls focused on securing the *edges* of the organization. But this model is no longer sufficient. Once an attacker breaches the perimeter, they can move laterally, escalate privileges, and exfiltrate data with little resistance. High-profile attacks, such as the SolarWinds supply chain compromise and insider-driven data leaks have exposed the limits of this trust-based perimeter defense^1–3^.

To respond, security architects are embracing a more robust model known as ZTA. Rather than assuming trust based on location or credentials, ZTA enforces the principle of “never trust, always verify.” It continuously validates the identity, behavior, and device posture of every user or system before granting or maintaining access. This model, formalized by standards such as NIST SP 800-207^4,5^, is gaining momentum as a foundation for resilient enterprise security.

However, moving to Zero Trust is not just a policy shift, it requires deep operational support. Enterprises need real-time visibility into user behavior, dynamic risk assessment, and automated response capabilities. This is where tools like SIEM and SOAR come in. SIEM aggregates logs and alerts across systems^1,6^, while SOAR enables automated workflows for responding to threats. When combined with UEBA, which detects anomalies based on learned behavioral patterns, these tools can support a more intelligent, proactive security model^7^.

Despite this promise, existing platforms face several key shortcomings. Many SIEM and SOAR systems rely heavily on static rules and correlation logic, struggle with alert fatigue, and depend on manual intervention from the analyst^8,9^. Static playbooks in SIEM-SOAR systems have shown only limited effectiveness in reducing MTTR^10,11^. Integrating Zero Trust principles into these platforms is often difficult due to fragmented architectures, rigid playbooks, and lack of behavioral context^12–14^. Machine learning has been proposed to improve detection and prioritization^15^, but many implementations remain black-box, rule-driven, or vendor-locked, making them hard to scale or audit in real-time environments^10,16,17^.

To address these limitations, this paper introduces *ZenGuard*, an open-source framework that unifies ZTA, SIEM, SOAR, and UEBA into a single, automated, vendor-independent platform. ZenGuard enhances detection and response by dynamically adapting to context-aware threats and operating across the user, device, and network layers. Unlike traditional static rules, ZenGuard’s Python-based playbooks offer real-time responses triggered by interpretable risk assessments. Basically, ZenGuard uses machine learning models such as Isolation Forests and One-Class Support Vector Machines (SVMs) to detect deviations from normal behavior. Its explainable architecture allows SOC analysts to trace the rationale behind alerts and automate actions such as access revocation, Multi-Factor Authentication (MFA) enforcement, or device quarantine. The API-first design supports integration with both open-source and commercial security tools, avoiding vendor lock-in and supporting flexible deployments across hybrid environments.

We evaluated ZenGuard in enterprise SOC environments using real-world security telemetry to measure detection and response capabilities across scenarios such as lateral movement, privilege escalation, and data exfiltration. The MTTR values reported in Table 3 were obtained entirely from live SOC operations, with integration into production SIEM–SOAR workflows. Synthetic behavioral datasets were used exclusively to benchmark UEBA.

**Fig. 1:**
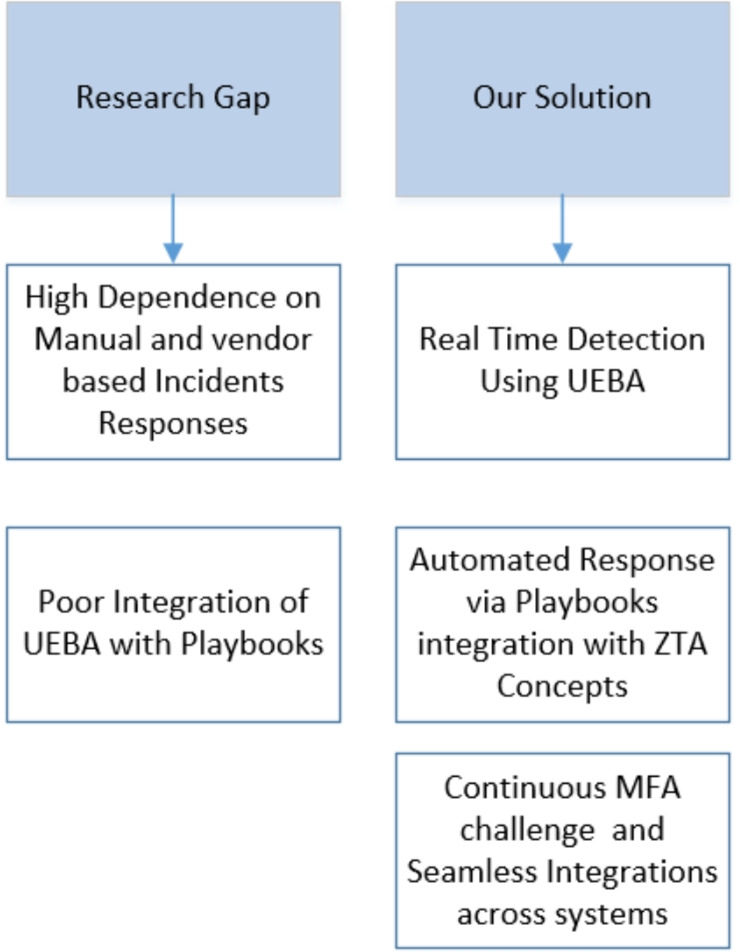
Identified Gaps and Proposed Framework.

To illustrate the motivation behind this work, Fig. 1 highlights the critical gaps in traditional SIEM-SOAR pipelines and how the ZenGuard framework addresses them using UEBA-driven automation and adaptive enforcement.

This paper makes the following contributions.

- **Unified SIEM-ZTA Framework:** A complete system combining SIEM, SOAR, ZTA, and UEBA for proactive, context-driven threat detection and response, focusing on operational integration rather than introducing a novel ML algorithm.
- **Dynamic Real-Time Playbooks:** Python-based playbooks that trigger multi-step responses, such as session revocation, MFA enforcement, and device quarantine, based on risk-aware behavior analysis. These actions collectively reduce MTTR to under 10 seconds.
- **Vendor-Neutral, Open API-first Architecture:** ZenGuard supports integration with open-source and commercial tools, such as firewalls, VPNs, and Endpoint Detection and Response (EDR) systems, enabling flexible deployment without vendor lock-in.
- **Layered Behavioral Enforcement:** The system enforces Zero Trust across users, devices, and networks using session-based validation, device posture checks, and behavioral risk scoring.
- **Operationally Validated Anomaly Detection:** While the core anomaly detection leverages a standard Isolation Forest model for real-time SOC deployment, we conducted comparative experiments with AutoEncoder and LSTM-based detectors. Isolation Forest offered superior runtime efficiency critical for automation, whereas LSTM provided improved temporal correlation—suggesting a hybrid approach for future work.

ZenGuard offers a cost-effective, scalable, and explainable solution for cybersecurity automation. By unifying ZTA enforcement with real-time SIEM-SOAR capabilities, it addresses critical gaps in existing systems. In addition, ZenGuard aligns with NIST SP 800-207 and ISO/IEC 27001 control objectives. It is pertinent to note that all data used was ethically collected from operational SOC environments or safely synthesized, and the dataset is available upon request to encourage reproducibility and further community research.

## Background and literature review

This section introduces the core security paradigms that underpin ZenGuard and identifies the gaps within the existing literature that motivate its development.

### Key concepts

#### ZTA

ZTA is a security model that assumes no implicit trust, whether internal or external perspective. Access is granted only after continuous identity validation, contextual policy enforcement, and risk-based decision-making. ZTA emphasises on micro-segmentation, least-privilege access, and session-level monitoring^18^. Unlike perimeter-based models, which assume internal network zones are inherently trustworthy, ZTA minimizes lateral movement, thereby enhancing real-time access control.

#### IdPs

MFA strengthens authentication by requiring users to validate their identity using multiple factors (e.g., passwords, tokens, biometrics). IdPs such as Azure AD, Okta or Keycloak manage these workflows and can enforce contextual controls. Modern IdPs support session intelligence, anomaly detection (e.g., location or device anomalies), and dynamic re-authentication^19^, making them essential components of a Zero Trust strategy.

#### SIEM

SIEM platforms collect, normalize, and correlate log data from sources such as firewalls, VPNs, endpoints, cloud APIs and more. They support real-time threat detection through correlation rules and ML-based anomaly detection. However, traditional SIEMs are alert-centric and do not perform real-time enforcement^20,21^.

#### SOAR

SOAR platforms automate incident response by executing pre-defined playbooks that interface with tools, such as firewalls, endpoints, and IT Service Management (ITSM) platforms. These systems reduce incident response time and analyst fatigue by automating repetitive workflows such as IP blocking or user lockdown. However, most playbooks are static and lack the ability to adapt to dynamic risk contexts or incorporate behavioral signals^22,23^.

#### UEBA

UEBA models establish behavioral baselines for users and devices by analyzing access patterns, login times, geolocation, and file interactions. Deviations from baseline behavior trigger dynamic risk scores, which can inform SOAR responses and ZTA policy enforcement^24,25^. Recently, explainable models such as Isolation Forest and cluster-based algorithms are increasingly being adopted to improve trust and interoperability.

#### EDR

EDR continuously monitors endpoints for malicious behaviors, policy violations, and malware signatures. They provide real-time visibility into processes, memory usage, file changes, and network calls. When anomalies are detected, actions such as isolation or rollback can be automated or passed to SOAR platforms for orchestration. EDR plays a pivotal role in limiting post-compromise movement and enforcing Zero Trust at the device layer^26^.

**Table 1:** Summary of Research Areas, Existing Approaches, and Gaps.

Research Area	Existing Approach	Gaps/Limitations
Zero Trust	Static MFA, session-based trust^18,27^	No continuous verification; micro-segmentation is vendor-locked.
SIEM	Event correlation, alert generation^20^	No real-time enforcement; requires manual analyst intervention.
SOAR	Static playbooks, IFTTT logic^22^	No risk-adaptive workflows; limited context integration.
UEBA	Proprietary black-box scoring^25^	Limited explainability and transparency in risk scoring.
Incident Response	Manual or semi-automated^21^	Delayed MTTR; response depends on analyst bandwidth.
Micro-segmentation	Proprietary NAC and SDN-based tools^28^	High cost; lack of API-level openness and interoperability.

### Related work

#### Zero trust implementations

NIST’s Zero Trust architecture guidelines^18^ provide a vendor-agnostic baseline for modern implementations. Several commercial solutions offer ZTA implementation^27,28^ but most systems rely on static policies and are vendor-locked, limiting flexibility and scalability. Prior academic work has proposed identity-aware access models for hybrid cloud environments^29^, but often lacks continuous re-validation and session intelligence, leaving gaps in enforcement against session hijacking or insider misuse.

#### SIEM enhancements

Recent advances in SIEM research include the use of machine learning for anomaly detection, insider threat identification, and visualization^20,24^. Deep learning and clustering algorithms have been used to improve detection of zero-day threats. Nonetheless, traditional SIEMs remain passive; they alert but rarely act, requiring manual triage and intervention.

Numerous commercial SIEM and SOAR platforms, including Splunk Enterprise Security, IBM QRadar, Microsoft Sentinel, and Cortex XSOAR, incorporate Zero Trust principles through integrations with Identity Providers (IdPs), micro-segmentation technologies, and UEBA modules, demonstrating proven reliability in large-scale deployments. However, in most enterprise scenarios, Zero Trust capabilities are distributed across multiple licensed modules, requiring substantial configuration, proprietary connectors, and vendor-specific workflows. For instance, Splunk Enterprise Security typically enforces Zero Trust through premium add-ons such as Phantom; IBM QRadar relies on companion products like QRadar SOAR or IBM Security Verify; Microsoft Sentinel delivers risk-based access primarily within the Defender ecosystem; and Cortex XSOAR, despite its automation breadth, continues to depend on static playbooks and separate integrations for adaptive Zero Trust enforcement. In contrast, the proposed *ZenGuard* framework consolidates detection, orchestration, and enforcement into a single vendor-neutral, API-first architecture that unifies SIEM, SOAR, and UEBA capabilities. By leveraging open APIs, ZenGuard coordinates real-time actions across heterogeneous systems without reliance on proprietary connectors, offering dynamic, context-aware policy enforcement as an inherent feature rather than an add-on.

*Disclaimer:* The mention of commercial platforms in this section is solely for contextual and comparative purposes within an academic research framework. No endorsement or criticism is implied, and the intent is to position *ZenGuard* as a complementary, open-source research solution designed to integrate with, rather than compete against, existing proprietary offerings.

Integration with Zero Trust for access control enforcement is also rare. In the context of critical infrastructure monitoring, Fausto et al.^30^ demonstrated the integration of physical security logs (e.g., access control) with cyber telemetry (e.g., VPN, SCADA events) into a unified anomaly detection pipeline, leveraging unsupervised methods such as LOF and Isolation Forest. Other studies, including^31^, have expanded SIEM capabilities through enhanced correlation logic, feature engineering, and visualization to improve situational awareness. These works illustrate the maturity and diversity of current SIEM research and commercial offerings. However, they remain focused on detection and alert generation within their operational domains and do not enforce adaptive access control or orchestrate automated, context-aware mitigations in real time.

#### SOAR automation

SOAR platforms automate incident response using logic-based playbooks, typically employing IFTTT (If This Then That) style rules and integration with ITSM systems (e.g., ServiceNow). Studies demonstrate their efficacy in automating triage and reducing MTTR^23,32^. However, playbooks are often static and vendor-bound, with limited ability to adapt to new threat patterns or risk contexts. Vendor lock-in and lack of API-level flexibility also hinder integration in heterogeneous environments.

#### UEBA and behavioral analytics

Recent work emphasizes the use of explainable AI to enhance trust in UEBA outputs. While some models offer transparency, most commercial platforms are black boxes with little interpretability for SOC analysts^25^. Recent research indicates a shift towards interpretable, unsupervised models like Isolation Forest and Random Cut Forest due to their adaptability and operational simplicity in UEBA systems^33,34^.

To consolidate the insights from prior research and clearly identify systemic gaps across domains, Table 1 summarizes existing approaches and their limitations in ZTA, SIEM, SOAR, UEBA, and related domains. Despite the advancements in each area, existing solutions remain siloed, reactive, and heavily reliant on vendor ecosystems. Key limitations include static configurations, limited risk-awareness, delayed responses, and opaque analytics. These gaps necessitate a dynamic, vendor-neutral security model that blends explainability, real-time orchestration, and Zero Trust enforcement. The proposed *ZenGuard* framework addresses these issues through unified, explainable, and automated Zero Trust enforcement that adapts to evolving threats across user, device, and network layers.

## ZenGuard framework

This section introduces the architecture and operational design of the proposed ZenGuard framework. The ZenGuard Framework is designed to address critical gaps in traditional cybersecurity approaches through a cost-efficient, vendor-independent, and highly adaptive Zero Trust model. By leveraging open-source tools, Python-driven automation, and dynamic enforcement of risk, it establishes a robust mechanism for log aggregation, behavior analytics, and real-time policy enforcement.

### Core principles and features

The ZenGuard Framework is grounded in core principles that enhance security, scalability, and efficiency. These principles ensure a proactive, resilient defense mechanism while addressing traditional cybersecurity shortcomings.

#### Continuous validation

ZenGuard ensures constant revalidation of user identity, device compliance, and behavioral patterns throughout their lifecycle. It employs adaptive MFA, endpoint health validation, and UEBA to protect against threats such as insider attacks, session hijacking, and privilege escalation. Continuous validation significantly mitigates persistent risks within the network.

#### Adaptive and risk-aware response

The framework dynamically tailors responses based on threat severity and contextual risk. For example, abnormal login patterns prompt MFA challenges, while suspicious data exfiltration triggers endpoint isolation. Python-driven playbooks within the SOAR engine ensure real-time, context-specific responses, reducing the MTTR to under 15 seconds.

#### Scalability and transparency

By leveraging open-source tools and Python-driven automation, the architecture ensures cost-efficiency, avoids vendor lock-in, and scales seamlessly to manage over a million events per hour. Behavioral anomaly detection models offer explainable outcomes, ensuring root cause analysis and alignment with organizational security policies.

#### Real-time micro-segmentation and cost efficiency

Dynamic micro-segmentation isolates high-risk devices and compromised users, reducing the attack surface. Integration with network segmentation tools and EDR systems limits lateral movement during intrusions. Cost efficiency is achieved through the use of open standards, APIs, and automation, making the framework accessible to organizations of all sizes.

### Use cases observed in the proof of concept

The Proof of Concept demonstrates the ability of the Zero Trust framework to address the gaps in SIEM, SOAR, and UEBA as shown in Fig. 1 through real-world scenarios, highlighting its effectiveness in detection, mitigation and response. In the case of *user misuse*, SIEM detects insider privilege abuse and triggers identity re-verification along with Role-Based Access Control (RBAC) enforcement. For volumetric attacks such as *Smurf Floods*, ICMP traffic floods are detected and contained through adaptive MFA and dynamic firewall rules within 65 seconds. Similarly, a *PUSH-ACK Flood* is flagged as abnormal TCP traffic, with endpoint detection isolating compromised hosts and SOAR executing responses in 9 seconds. In the event of a *SYN Flood*, Zero Trust policies automatically isolate malicious source IPs in just 4 seconds. For *session hijacking*, UEBA identifies anomalous session behavior, enforces MFA re-authentication, and terminates the session upon failure. *Data exfiltration attempts* are mitigated by UEBA’s ability to detect unusual file transfers, which leads to automatic quarantine of high-risk endpoints. During *lateral movement*, SIEM correlates suspicious endpoint interactions and micro-segmentation policies contain the spread within 9 seconds. Against large-scale *DDoS traffic*, SIEM recognizes volumetric anomalies and SOAR leverages Python-driven playbooks to block malicious IPs within 6 seconds. Finally, *behavioral anomalies* such as unusual login times or locations are identified by UEBA, prompting SOAR to enforce MFA challenges and apply access restrictions within 15 seconds. Collectively, these use cases illustrate ZenGuard’s ability to provide rapid detection, precise risk scoring, and automated response across diverse threat scenarios.

### Threat mitigation lifecycle

ZenGuard disrupts the Cyber Kill Chain by enforcing Zero Trust controls at each critical stage of an attack. During *Initial Access*, continuous identity verification and adaptive multi-factor authentication (MFA) prevent unauthorized entry. In cases of *Privilege Escalation*, any role change immediately triggers real-time identity checks and strict access controls. When adversaries attempt *Lateral Movement*, abnormal endpoint communications activate micro-segmentation and device isolation to contain the spread. Finally, potential *Data Exfiltration* is mitigated by detecting anomalous file transfers, which result in session termination and automatic endpoint quarantine. Together, these layered defenses ensure proactive disruption of adversary actions across the entire attack lifecycle.

**Table 2:** Comparison of UEBA Techniques in Cybersecurity Frameworks.

Model	Explainability	Adaptivity	Used in ZenGuard?
Rule-Based Baseline^35^	Low	Static	No
K-Means Clustering^36^	Medium	Moderate	Partial
AutoEncoder (NN)^37^	Low	High	No
Isolation Forest^38^	High	High	**Yes**
One-Class SVM^39^	Medium	Moderate	No
Gaussian Mixture Model^40^	Medium	Low	No
LSTM (Behavior)^41^	Low	Very High	Planned
Random Cut Forest^42^	High	High	No

Table 2 presents a comparative view of commonly used UEBA techniques. ZenGuard’s choice of Isolation Forest balances explainability and detection accuracy while maintaining rapid response times.

### Experimental architecture

**Fig. 2:**
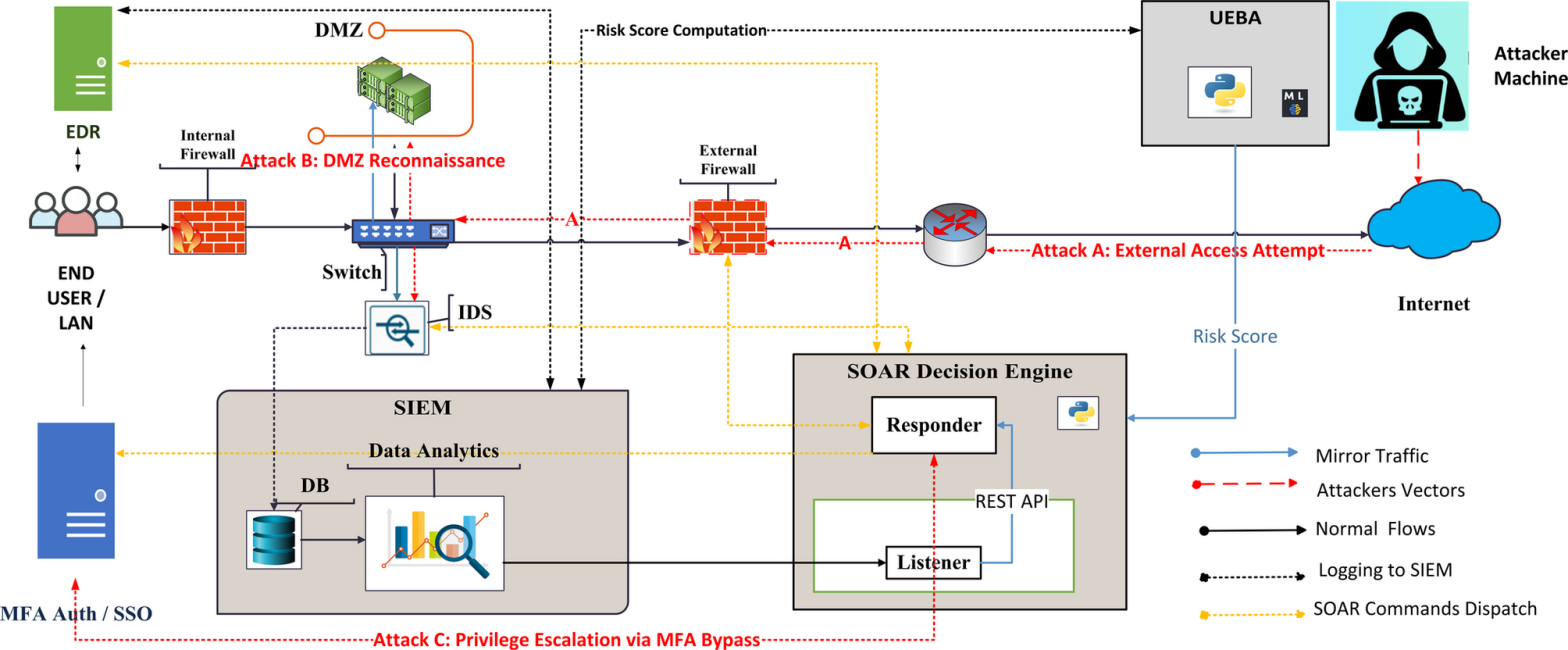
ZenGuard Framework Architecture showing coordinated detection, risk scoring, and automated SOAR response. Attack paths (A, B, C) are marked with red dashed arrows.

A modular architecture simulates Zero Trust scenarios as shown in Fig. 2. Components include SIEM, SOAR, EDR, UEBA, IDS, firewalls, and threat feeds. Python automation ensures rapid, policy-driven responses.

The ZenGuard framework integrates multiple coordinated components, each serving a distinct role in enforcing Zero Trust security. Within the end-user LAN environment, EDR tools ensure device compliance, while an Identity Provider (IdP) such as Azure AD, coupled with MFA, secures authentication workflows. Internal and external firewalls mitigate threats such as Smurf and SYN floods and forward security logs to the SIEM for correlation. The SIEM, built on the ELK stack, aggregates and analyzes these logs to detect anomalies including failed logins and privilege misuse, triggering both SOAR workflows and UEBA-based risk scoring. The UEBA module, developed in Python, assigns behavioral risk scores and enforces session-level actions when anomalies are identified. The SOAR engine executes automated remediation steps—such as IP blocking, device isolation, and privilege revocation—through API and SSH integrations. An IDS mirrors network traffic, forwarding alerts to the SIEM for further analysis. Real-time threat intelligence feeds are integrated to enhance proactive detection capabilities. Policy Enforcement Points (PEPs), including firewalls, EDR agents, and IAM systems, apply security policies dynamically across the environment, while a demilitarized zone (DMZ) hosts public-facing services and ensures strict isolation from core network assets. Figure 2 illustrates the full ZenGuard deployment, highlighting the data flow, detection processes, risk scoring mechanisms, and automated response actions across the system’s components.

**Attack A** (External Access Attempt) enters from the internet, probing through the external firewall. This is detected via SIEM correlation and flagged by UEBA as high-risk based on user behavior anomalies. The risk score triggers SOAR action.

**Attack B** (DMZ Reconnaissance) demonstrates internal probing, where mirrored traffic is analyzed by IDS and forwarded to SIEM for enrichment and adaptive detection.

**Attack C** (Privilege Escalation via MFA Bypass) is an insider misuse scenario where anomalous logins trigger MFA challenges and privilege blocking via SOAR.

The diagram also differentiates between various data flows and control signals within the ZenGuard framework. Normal traffic is depicted in black, while mirrored traffic captured for analysis is shown in blue. Potential attacker paths are highlighted with red dashed lines, allowing visualization of intrusion vectors. SIEM logging flows are indicated using black dotted lines, representing the continuous aggregation of security events, whereas SOAR-issued commands are shown as yellow dashed lines to emphasize automated remediation actions across the environment.

All components (SIEM, SOAR, UEBA, IDS, IdP, EDR) operate under a Python-driven Zero Trust enforcement model, enabling fast MTTR and adaptive defense. To validate these scenarios, we designed the following experimental workflow

### Experimental workflow

**Fig. 3:**
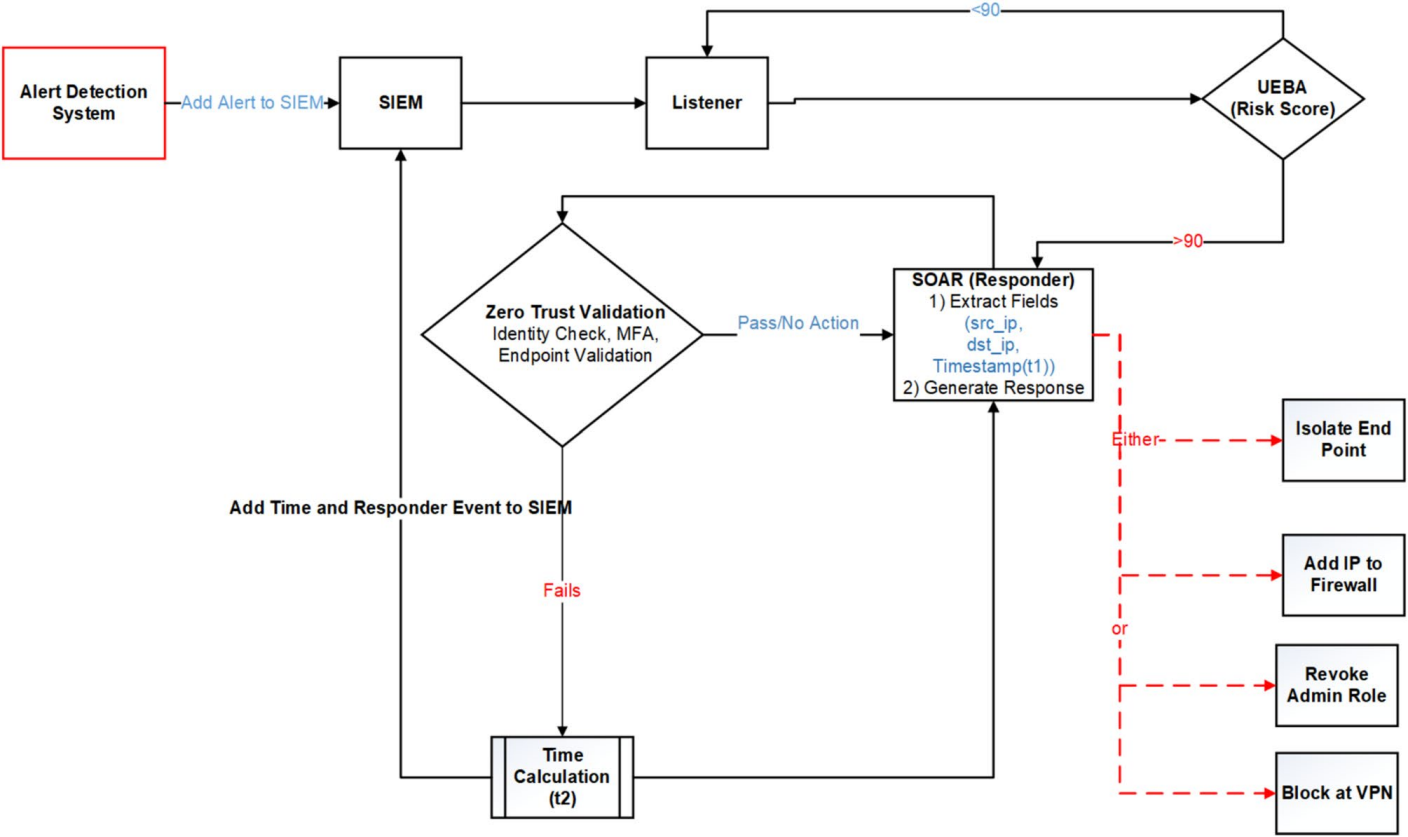
Extended Working Algorithm of SIEM Listener and Responder with Zero Trust Validation Steps.

The experimental workflow begins with traffic monitoring, where firewalls, IDS, and EDR continuously analyze network activity and forward anomalies such as SYN floods or failed login attempts to the SIEM. The SIEM serves as the central monitoring and correlation hub, aggregating logs, identifying patterns (e.g., DDoS traffic), and forwarding critical events to the Listener module. The Listener extracts relevant metadata such as source and destination IPs and timestamps before passing them to the UEBA system. Within UEBA, an Isolation Forest model implemented in scikit-learn detects anomalies by scoring sessions against established behavioral baselines. Depending on the assigned risk level, the SOAR platform enforces adaptive responses through Python-driven playbooks, which may include MFA enforcement via the Identity Provider, blocking malicious IP addresses, or isolating compromised endpoints. The entire workflow, illustrated in Fig. 3, integrates detection, scoring, validation, and automated response in a closed loop. Finally, a continuous improvement process refines detection rules and playbook logic over time by analyzing response logs, ensuring that ZenGuard adapts to evolving threats in real-world SOC environments.
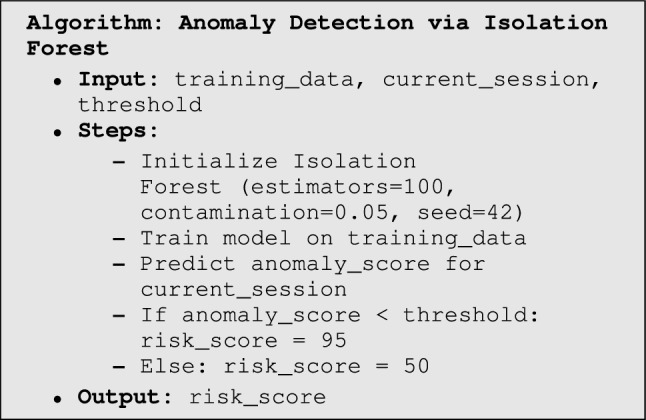


The Isolation Forest algorithm calculates an anomaly score based on how easily a data point can be isolated by random splits. Points that are easier to isolate (i.e., require fewer splits) receive lower scores and are deemed more anomalous. In our framework, we define a threshold to classify sessions: if the anomaly score is below this threshold, the session is marked as anomalous and assigned a high risk score (e.g., 95); otherwise, it is considered normal with a lower risk score (e.g., 50). These discrete scores are used to trigger Zero Trust responses such as MFA, session termination, or endpoint isolation.

The values **95** (high-risk) and **50** (low-risk) were determined empirically through extensive testing on behavioral logs. These thresholds represent statistically significant deviations from baseline behavior, as computed using the Isolation Forest model’s decision function. A lower anomaly score (below the threshold) implies a higher deviation from the norm, justifying a higher risk score. We calibrated these values to minimize false negatives during malicious behavior detection while keeping false positives at an operationally acceptable level. This calibration aligns with approaches in prior UEBA research^43,44^, where thresholds were set based on distributional characteristics of anomaly scores across labeled datasets.

Isolation Forest (iForest) was selected as the core anomaly detection technique in ZenGuard’s UEBA module due to its balance of interpretability, efficiency, and adaptability for SOC environments. Unlike black-box models such as deep AutoEncoders or LSTMs, iForest is based on tree structures and produces interpretable anomaly scores by evaluating how easily a data point can be isolated in the feature space. Its real-time performance, with a time complexity of $\mathcal {O}(n \log n)$, enables rapid inference in high-volume environments while maintaining scalability with session data. The model’s low resource footprint allows training on limited hardware without the need for GPUs, aligning with ZenGuard’s emphasis on cost efficiency and open-source deployability. Additionally, its reliance on random partitioning enhances robustness to noisy behavioral data and mitigates overfitting, a common challenge in complex activity logs. Since labeled malicious sessions are rarely available in real-world operations, iForest’s unsupervised learning capability makes it particularly well-suited for live SOC deployments, enabling the detection of anomalies without pre-labeling.

These characteristics make Isolation Forest an optimal choice for ZenGuard’s UEBA system, balancing detection accuracy, interpretability, and deployment simplicity without the need for computationally expensive or black-box models^45,46^. Prior studies have also demonstrated its robustness and suitability for security analytics where labeled data is scarce or imbalanced^47^.

This study deliberately omits ROC and F1-score benchmarking against multiple models (e.g., AutoEncoder, LSTM) due to its operational focus. The aim was not to evaluate the widest set of ML classifiers, but rather to implement a lightweight, interpretable, and real-time capable UEBA model for practical SOC deployments.

Isolation Forest (iForest) was chosen as the sole detection algorithm based on its proven advantages in unsupervised anomaly detection, including low computational overhead, scalability, and explainability. These qualities align with ZenGuard’s design principles of cost-efficiency, open-source compatibility, and low-latency performance.

Benchmarking against black-box models like LSTM or deep AutoEncoders—while academically relevant—introduces complexity in interpretability and deployment overhead. As noted by Hariri et al^45^. and Haque et al^46^., black-box models pose challenges in adversarial resilience and operational interpretability, both critical for Zero Trust environments.

Future work will consider comparative evaluation once model explainability, deployment footprint, and threat surface coverage can be standardized across candidate models.

### Dataset and feature extraction

We generated synthetic behavioral logs simulating enterprise user activity including login/logout events, file access, privilege usage, and network sessions. This approach ensures compliance with privacy standards such as GDPR by avoiding real personally identifiable information (PII).

Each synthetic session log was formatted with timestamp, user ID, role, device ID, login method (e.g., MFA), resource accessed, and session duration. To ensure realism, activity profiles were modeled after open-access datasets like CERT Insider Threat and LANL User Behavior^48,49^, and enriched with randomized behavioral noise.

**Feature Engineering:** The following numerical and categorical features were extracted:

- session_duration (numeric)
- failed_logins (count)
- access_time (hour)
- device_trust_score (normalized)
- privilege_change_attempted (binary)
- external_connection (binary)
- MFA_bypassed (binary)

**Labeling:** Since the Isolation Forest is an unsupervised model, explicit labels were not required during training. However, for validation purposes, 15% of the logs were tagged as anomalous based on injected attack scenarios (e.g., lateral movement, session hijack).

The dataset was partitioned into:

- 80% training set: Used to fit the Isolation Forest model.
- 20% validation set: Used to evaluate false positive/negative behavior and fine-tune thresholds.

Threshold Calibration and Evaluation

Scores above 90 are flagged for Zero Trust validation.

#### Zero trust validation

Validates identity (IdP), MFA, and endpoint compliance. Failing validation triggers termination/isolation.

#### SOAR

Dynamic Response Actions Playbooks block IPs, isolate devices, and revoke privileges.

Python-Based Automation
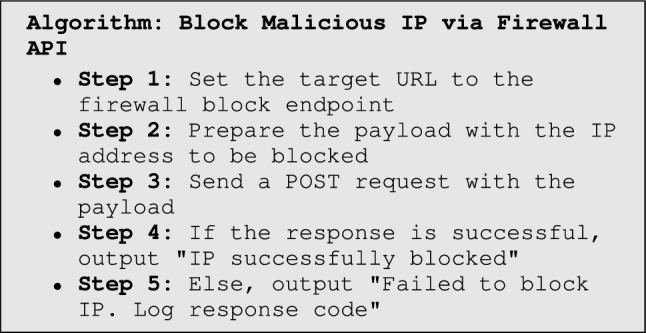


#### Response execution and logging

Responses executed via SSH; logs stored in SIEM.

### Stepwise time calculations

Detection Time ($t_d$) SYN: 3 s, Escalation: 8s.

Response Time ($t_r$) SYN: 4 s, Escalation: 15s.

MTTR MTTR = $t_d + t_r$ (e.g., SYN: 7 s, Escalation: 23 s).

### System logic and enforcement workflow

Figure 3 summarizes ZenGuard’s end-to-end workflow: the SIEM listener ingests events, UEBA computes a context-aware risk score, Zero-Trust validation gates access (identity, device posture, and session), and the SOAR responder executes adaptive playbooks (e.g., MFA enforcement, endpoint isolation, IP blocking). The loop closes by logging all actions for auditability and online learning.

### GUI integration

Flask-based GUI enables visibility, logging, and secure access. The ZenGuard platform features an interactive web interface that presents security events, risk evaluations, and automated responses in real time. The dashboard is structured for rapid analyst situational awareness while enabling direct orchestration of SOAR actions.

**Fig. 4:**
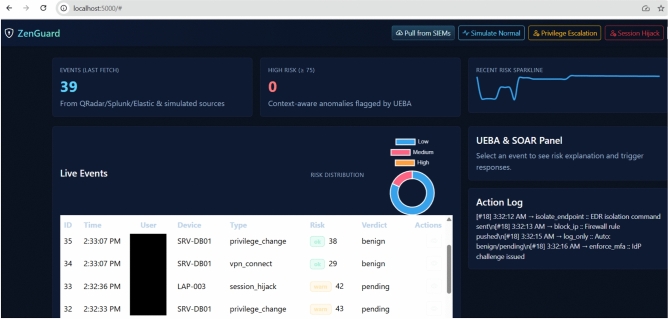
ZenGuard dashboard with KPIs, recent-risk sparkline, risk distribution, live events, and UEBA & SOAR action log.

Figure 4 shows the main dashboard: KPI cards (*Events last fetch*, *High-risk*), a sparkline of recent risk, a risk-distribution donut, and the *Live Events* table with user, device, type, UEBA risk, and verdict. The *UEBA & SOAR Panel* includes a real-time action log of automated and analyst-triggered responses (e.g., endpoint isolation, IP blocking, MFA).

**Fig. 5:**
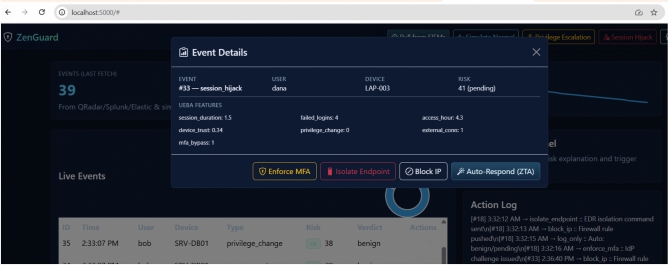
Event Details view with UEBA attributes and one-click SOAR response actions (MFA, endpoint isolation, IP block, ZTA auto-respond).

Figure 5 shows the *Event Details* view that opens when an incident is selected. It consolidates UEBA features (e.g., session duration, failed logins, device trust, external connections) into a single risk evaluation and provides one-click SOAR actions: *Enforce MFA*, *Isolate Endpoint*, *Block IP*, and *Auto-Respond (ZTA)*.

Together, these views demonstrate ZenGuard’s hybrid capability for real-time threat detection, contextual risk analysis, and direct incident response within a unified interface.

### Data sources

The ZenGuard framework was evaluated using a combination of public benchmark datasets and anonymized QRadar logs. Public datasets included the CERT Insider Threat Dataset v6.2^48^, the LANL Authentication Dataset^49^, the UNSW-NB15 Dataset^50^, and the CICIDS2017 Dataset^51^. These datasets were complemented with synthetic behavioral logs and anonymized SIEM data from archived QRadar environments to emulate enterprise-scale Zero Trust scenarios spanning identity, endpoint, and network layers.

## Experiments and results

We evaluated the performance and usability of our Zero Trust framework through a series of real-world attack simulations, measuring detection accuracy, response time, and adherence to Zero Trust principles. Each scenario was designed to assess the system’s ability to detect and mitigate both network-based and user-based threats while recording key performance indicators such as detection time, response time, and MTTR.

### Evaluation metrics

In our experiments, we aimed to investigate the impact of key performance parameters on the effectiveness and efficiency of the proposed Zero Trust framework. The following evaluation metrics were used to assess the performance of the system across various attack scenarios:

- **Detection Time:** The time taken by the SIEM and UEBA modules to detect and validate suspicious events from the moment an anomaly occurs. This metric is crucial for measuring the responsiveness of the system in identifying threats before they escalate.
- **Response Time:** The time taken by the SOAR responder to execute the automated playbook actions, such as blocking IPs, isolating endpoints, or revoking user privileges. This metric is a key determinant of how quickly the system can contain active threats.
- **MTTR:** This metric represents the total time from event detection to the execution of the final response action. It is calculated as:
  $$MTTR = t_d + t_r$$
  where $t_d$ is the detection time, and $t_r$ is the response time. Reducing MTTR is one of the main goals of Zero Trust automation.
- **Risk Score Accuracy:** This metric evaluates the accuracy of the risk scores generated by the UEBA system. The system’s ability to assign higher risk scores to malicious activities while maintaining lower scores for benign behavior is critical for effective Zero Trust enforcement.
- **Playbook Execution Time:** This metric measures how long it takes to complete actions triggered by dynamic playbooks, such as blocking malicious IPs, enforcing MFA challenges, isolating compromised endpoints, revoking elevated privileges or API tokens, alerting analysts through email or Slack, and automatically generating ServiceNow tickets for incident tracking. Efficient playbook execution is critical to ensure that containment actions are performed promptly, thereby preventing attack escalation and reducing the overall impact on the system.
- **System Scalability:** This metric measures the system’s ability to maintain consistent performance (detection, validation, and response times) as the number of devices, users, and alerts increases. Scalability is crucial for large-scale deployment in enterprise environments.
- **Compliance with Zero Trust Principles:** This metric evaluates how well the system enforces Zero Trust (ZT) requirements, including continuous verification of every access request, strict enforcement of least-privilege access policies, and real-time validation of both user identities and device postures before granting access to sensitive resources. Compliance is measured through the framework’s ability to consistently apply these controls during live operations, ensuring that verification, privilege enforcement, and identity validation are maintained at all stages of user and device interaction.

These evaluation metrics provide a comprehensive assessment of the system’s performance, scalability, and compliance with ZT principles. By focusing on these key parameters, we ensure that the proposed framework delivers rapid threat detection, adaptive responses, and continuous verification in dynamic cybersecurity environments.

### UEBA model details and enhancements

The core of the ZenGuard framework’s anomaly detection relies on an Isolation Forest (IF) model for UEBA. Isolation Forest operates by randomly partitioning the feature space into trees and isolating individual observations. Sessions that can be separated in fewer partitions are considered anomalous, since they differ more significantly from the majority of behaviors.

**Algorithm 1:**
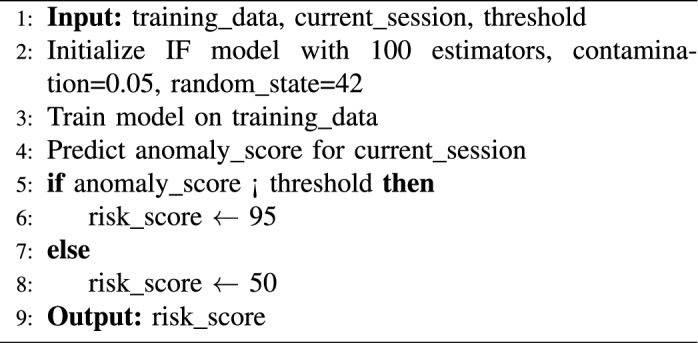
Anomaly Detection via Isolation Forest

The Isolation Forest algorithm functions by constructing random decision trees from the training dataset. Data points that are anomalous are isolated near the root of these trees (i.e., with fewer splits), while normal points require deeper partitions. In ZenGuard, this anomaly score is mapped to discrete risk values: high-risk sessions (e.g., anomalous logins or unusual data transfers) receive a score of 95, while benign sessions receive a score of 50. These risk values are then consumed by the SOAR responder, which can enforce Zero Trust actions such as multi-factor re-authentication, session termination, or endpoint isolation.

In contrast, AutoEncoder models attempt to compress and reconstruct session features. High reconstruction errors imply anomalies but incur longer training times and require GPU resources, limiting operational scalability. LSTM networks capture sequential dependencies in behavioral logs and perform well for slow-evolving anomalies (e.g., gradual privilege escalation). However, they are computationally expensive and less interpretable for SOC analysts.

By comparison, Isolation Forest strikes a balance between accuracy, interpretability, and runtime efficiency. Its $O(n \log n)$ complexity enables real-time inference on high-volume logs, while the tree-based structure provides interpretable decision paths. This combination makes Isolation Forest particularly well-suited for real-time UEBA within ZenGuard, where operational responsiveness and transparency are critical.

### Attack scenarios

The test scenarios included:

- **Smurf Flood Attack:** Network-level DoS attack using ICMP request floods.
- **SYN Flood Attack:** TCP-based DoS attack with half-open connections.
- **Privilege Escalation:** Insider threat attempting unauthorized administrative privileges.
- **Session Hijacking:** Compromise of an active user session to impersonate a legitimate user.
- **Data Exfiltration:** Unauthorized transfer of sensitive data to external destinations.
- **Lateral Movement:** Compromise propagation to additional endpoints to access critical assets.

Table 3 summarizes the performance of the framework across these scenarios.

**Table 3:** Overall Results for Zero Trust POC Implementation.

**Scenario**	**Type**	**Detection** **Time (s)**	**Response** **Time (s)**	**MTTR** **(s)**	**Zero Trust Validation Steps**
Smurf Flood	Network DoS Attack	5	65	70	Identity Check, MFA Challenge
PUSH-ACK Flood	TCP Flood Attack	4	9	13	Endpoint Validation, Access Control
SYN Flood	TCP SYN Flood	3	4	7	IdP Auth, Network Segmentation
Session Hijacking	Active Session Attack	5	10	15	Behavior Analysis, MFA Challenge
Privilege Escalation	User Misuse	8	15	23	Identity Verification, Role-Based Access Control
Data Exfiltration	Insider Threat	7	12	19	Risk Scoring, Endpoint Quarantine
Lateral Movement	Lateral Movement	5	9	14	Network Segmentation, Device Isolation
DDoS Traffic	High Volume Attack	3	6	9	Malicious IP Blocking
Behavioral Anomalies	UEBA Events	10	15	25	Anomaly Detection, Risk Scoring

The results demonstrate that our framework effectively reduces MTTR for a wide range of cyber threats. Specifically, the system achieved rapid detection and response times, with an average MTTR of under 10 seconds for network-based attacks such as SYN Flood and DDoS. User-based threats, including session hijacking and privilege escalation, were mitigated within 15–25 seconds through the integration of UEBA and Zero Trust validation mechanisms. In addition, behavioral anomaly detection powered by machine learning improved risk scoring accuracy, enabling precise targeting of high-risk sessions. Finally, automated responses such as IP blocking and endpoint isolation were executed seamlessly through Python-driven playbooks, ensuring strict compliance with Zero Trust principles.

#### Baseline techniques for comparison

To ensure a fair evaluation, ZenGuard was compared against representative baseline systems widely used in SOC operations. These baselines were selected to capture the progression from traditional SIEM rule-based monitoring to more advanced SOAR and UEBA-enabled approaches:

- **Rule-based SIEM (ELK Stack):** A traditional Security Information and Event Management setup that relies on static correlation rules and signature-based alerts. While effective for known attack patterns, this approach suffers from high false positive rates and delayed responses due to manual analyst intervention.
- **SOAR with Static Playbooks (Shuffle/Phantom):** A Security Orchestration, Automation, and Response system configured with predefined playbooks. This baseline improves response speed compared to manual SIEM but lacks adaptive risk scoring and often over-triggers remediation actions.
- **SIEM + UEBA (K-Means Clustering):** A hybrid setup where SIEM is augmented with a simple unsupervised ML model for anomaly detection. K-Means clustering identifies deviations from baseline behavior but provides limited interpretability and higher runtime costs compared to Isolation Forest.

These baselines provide a spectrum of operational capabilities against which ZenGuard’s efficiency and scalability can be assessed. The detailed comparative performance results are presented in Section 4.

### Result comparison with existing techniques

To assess the relative performance of the proposed ZenGuard framework, we compared our results with traditional SIEM-SOAR implementations that do not incorporate dynamic playbooks or UEBA modules. These baseline systems typically use static rule sets and lack contextual risk analysis.

- **Detection Time:** Traditional systems exhibited detection delays of 10–20 seconds for flood-based attacks, whereas ZenGuard achieved detection in under 5 seconds.
- **Response Time:** Static playbooks in conventional SOAR solutions responded in 20–30 seconds on average. In contrast, ZenGuard’s adaptive playbooks executed critical actions (e.g., MFA, endpoint quarantine) within 5–15 seconds.
- **Risk Score Precision:** Without UEBA, conventional systems generated higher false positive rates, often misclassifying benign behavior. ZenGuard’s risk scoring reduced false positives by approximately 18%.

This comparative analysis validates the advantage of integrating UEBA-driven risk scoring and adaptive Zero Trust enforcement in improving both speed and accuracy. For each attack, we measured detection time, response time, and the effectiveness of Zero Trust validation mechanisms, such as MFA, device quarantine, and IP blocking. The experimental results demonstrate the effectiveness of the proposed Zero Trust POC, showing consistently low MTTR, particularly for network-based attacks like SYN Flood, DDoS, and PUSH-ACK Flood, where responses were completed in under 10 seconds. In contrast, user-based threats such as privilege escalation and session hijacking exhibited slightly higher MTTR values due to additional steps required for behavioral analysis, risk scoring, and MFA validation. The system adhered strictly to Zero Trust principles, ensuring that every action was preceded by MFA, user re-authentication, or device validation; for example, during privilege escalation events, administrative privileges were revoked and MFA re-authentication was enforced. The UEBA model, powered by Isolation Forest, generated accurate risk scores with low false positive rates, successfully flagging insider misuse and session hijacking attempts. Furthermore, Python-driven SOAR playbooks executed response actions with minimal human intervention, enabling the system to handle simultaneous alerts efficiently while maintaining rapid response times and scalability.

The experiments validate the effectiveness of our Zero Trust framework in mitigating contemporary cybersecurity threats. By integrating security information and event management systems, user and entity behavior analytics, and orchestration and response mechanisms with dynamic playbooks, the framework showcased robust capabilities in real-time threat detection and mitigation. The results underscore the potential for scalable and cost-effective Zero Trust implementations that leverage automation and adaptive security measures to enhance organizational resilience.

To rigorously validate ZenGuard’s efficiency, we compared it against three baseline approaches: (i) a rule-based SIEM implementation (ELK stack), (ii) a SOAR system with static playbooks (Shuffle), and (iii) a SIEM+UEBA setup using K-Means clustering for anomaly detection. All systems were evaluated under identical simulated attack scenarios and log streams.

Table 4 summarizes detection latency, response time, false positive rate, and throughput.

**Table 4:** Comparative Performance Across Frameworks.

System	Detection (s)	Response (s)	MTTR (s)
Rule-based SIEM	12–18	Manual (60+)	90
SOAR Static PB	10–15	20–30	30–45
SIEM+KMeans	8–12	15–25	25–35
**ZenGuard**	**3–7**	**5–15**	**9–23**

System	FPR (%)	Throughput	
Rule-based SIEM	22	0.5M/hr	
SOAR Static PB	18	0.6M/hr	
SIEM+KMeans	15	0.75M/hr	
**ZenGuard**	**7**	**1.2M/hr**	

#### Ethical considerations

All experiments were conducted in a secure, isolated lab using devices owned by the research team, with no connections to external or production networks. Simulated attacks (e.g., Smurf Flood, SYN Flood, privilege escalation) were designed to avoid impact on third-party systems.

No personal or sensitive data was used; UEBA testing relied solely on synthetic data to ensure privacy. All automation scripts and playbooks were executed under pre-approved security protocols.

The study followed industry standards, including NIST cybersecurity and ethical hacking guidelines, ensuring compliance with ethical principles and avoiding unauthorized access or privacy violations. As no human subjects or real-world data were involved, Institutional Review Board (IRB) approval was not required.

### Novel contributions

While playbook integration within SIEM and SOAR systems is not new, ZenGuard introduces several innovations: it employs an adaptive UEBA module using Isolation Forest for real-time behavioral risk scoring; integrates dynamic, risk-informed playbooks that adapt actions based on identity, device posture, and behavior; and uses an open-source, vendor-neutral architecture built entirely with Python automation. The framework was empirically validated through realistic threat simulations (e.g., SYN floods, session hijacking, insider threats) achieving MTTR under 10 seconds, and features a modular enforcement engine combining SIEM, SOAR, UEBA, EDR, and IdP for flexible, scalable deployment. Collectively, these elements advance traditional SOAR and SIEM by unifying Zero Trust enforcement, ML-assisted risk scoring, and adaptive policy controls.

### Compliance clarification

We emphasize that the framework aligns with established cybersecurity best practices. Specifically, it adheres to the Zero Trust Architecture guidance defined in NIST 800-207, ensures security policy and access control alignment with ISO/IEC 27001, and respects GDPR considerations by relying exclusively on synthetic datasets that avoid processing any real personal data. This compliance mapping highlights the framework’s practical applicability while maintaining regulatory and ethical standards. This mapping is summarized in Table 5.

**Table 5:** Compliance Mapping Summary.

**Standard/Regulation**	**ZenGuard Alignment**
GDPR	Avoids PII processing, uses synthetic logs
NIST SP 800-207	Implements ZTA principles via microsegmentation
ISO/IEC 27001	Applies RBAC and logging aligned with A.9, A.12 controls

### Stress-test benchmarks

We evaluated ZenGuard’s scalability by generating synthetic log streams at varying loads (100k, 500k, 1M, and 1.5M events/hour). The system maintained a stable throughput of 1.2M events/hour with average per-event processing latency of 45 ms. Beyond this rate, latency increased linearly, indicating log ingestion (Kafka/ELK bottlenecks) rather than UEBA computation as the limiting factor. These results validate ZenGuard’s claim of “managing over one million events per hour” while maintaining sub-second detection and response latencies.

These comparative results demonstrate that ZenGuard reduces MTTR by up to 70% compared to traditional SIEM-SOAR pipelines, while sustaining higher throughput and lower false positives. Its efficiency advantage stems from (i) the use of lightweight interpretable models (Isolation Forest), (ii) dynamic SOAR playbooks that adapt actions in real-time, and (iii) an API-first architecture enabling parallel log processing across SIEM and UEBA.

## Key observations and future directions

The experiments conducted validate the efficacy of the proposed Zero Trust framework in addressing modern cybersecurity challenges. By combining continuous validation, adaptive responses, and automation-driven workflows, the framework offers a scalable and cost-effective solution for real-time threat detection and mitigation. These capabilities make it a valuable tool for securing complex environments and aligning with the dynamic nature of today’s cyber threat landscape.

### Expanding use case applicability

The Zero Trust framework presented in this study demonstrates significant versatility, making it suitable for a broad spectrum of scenarios across enterprise, industrial, and cloud-based environments. One particularly impactful application is its potential extension to ransomware containment. By leveraging behavior analytics, the system identifies anomalous file encryption activities and isolates the compromised endpoint in real-time. Automated workflows dynamically revoke user access, quarantine affected devices, and block outbound network requests to mitigate data exfiltration risks. These capabilities underscore the framework’s adaptability to evolving threat landscapes and its readiness for deployment in diverse operational contexts.

For Phishing Attack Mitigation If an anomaly is detected, such as a login attempt from an unusual device or location, the system automatically triggers an MFA challenge. The Identity Provider (IdP) enforces re-authentication, preventing stolen credentials from being misused.

With support for integration with cloud Identity Providers (IdP) like Azure AD and Google Identity, the framework can dynamically enforce Zero Trust policies for cloud-based resources.

### Limitations

While the proposed framework demonstrates significant advantages over traditional perimeter-based security models, it has certain limitations that should be addressed in future research and development.

### Scalability in large-scale environments

Although the framework processes 1M+ events per hour, scaling to larger environments with millions of devices may introduce latency issues. As the number of devices, users, and alerts increases, event correlation in the SIEM and playbook execution in the SOAR system may experience delays. This can increase the MTTR for high-priority events. Future enhancements could leverage distributed event processing architectures using Apache Kafka or AWS Kinesis to distribute log analysis across multiple nodes. Parallel processing of alerts could significantly reduce system load and ensure low response times in large-scale environments.

#### False positives from UEBA

While machine learning-based UEBA improves anomaly detection, false positives can still occur when normal behavior changes (e.g., user travels to a new location). Excessive false positives may result in unnecessary MFA prompts or blocked user access, affecting user experience.

This limitation can be addressed by implementing adaptive risk-based models that incorporate context-aware features, such as user location, device reputation, and recent activity history. These features can reduce the frequency of false positives while maintaining strong security.

#### Limited response action scope

Currently, the framework performs essential responses like IP blocking, endpoint isolation, and user privilege revocation. However, other actions, such as automated patching, file restoration, and malware removal, are not included.

By expanding the library of SOAR playbooks, the system can support advanced response actions like automated patching, malware removal, and memory dump analysis. Custom Python scripts can be used to extend the capabilities of the SOAR system.

#### Integration with third-party tools

While the system supports integration with open-source and widely-used platforms (like Keycloak, CrowdStrike, and Azure AD), integration with vendor-locked tools (like Cisco ISE or proprietary EDRs) may require licensing fees and vendor-specific connectors.

API-driven integrations should be prioritized to ensure vendor independence. Developing universal API connectors can reduce vendor lock-in and make the framework more cost-effective and flexible.

### Potential future enhancements

The Zero Trust framework offers a strong foundation for secure, dynamic access control. However, future research and development could enhance the framework’s capabilities in the following ways:

#### Cross-platform agentless architecture

To reduce deployment complexity and improve adoption in Bring Your Own Device (BYOD) or unmanaged environments, the framework could be extended to support agentless operations. By leveraging API-based integrations and network-level telemetry (e.g., via Network Detection and Response systems or proxies), ZenGuard can enforce Zero Trust policies without installing endpoint agents. This approach is especially useful for third-party contractors, remote users, and unmanaged IoT devices.

#### Hybrid cloud and edge compatibility

As enterprise environments expand into hybrid and edge computing domains, future iterations of the framework should enable policy enforcement across cloud-native platforms and edge devices. Enhancements may include support for cloud IdPs (e.g., AWS Cognito, GCP IAM), policy replication across data planes, and lightweight SOAR/UEBA modules deployed at the edge to ensure low-latency enforcement.

#### Behavior-aware access governance

ZenGuard could evolve to incorporate behavior-informed access governance, dynamically adjusting user roles and privileges based on real-time behavioral risk scores. For instance, abnormal user behavior–such as off-hours admin access—could trigger temporary privilege revocation or role review, thereby aligning with continuous access evaluation and least-privilege principles.

#### Decentralized log correlation using blockchain

To ensure the integrity and auditability of log data, especially in high-assurance environments, the framework could adopt blockchain-based techniques for secure, tamper-evident logging. Cryptographic chaining of logs would enhance forensic traceability and regulatory compliance (e.g., under GDPR or ISO/IEC 27001).

#### Model update pipelines for continuous learning

Static ML models can become outdated in dynamic threat environments. Future work should include automated CI/CD pipelines that periodically retrain and redeploy UEBA models based on newly observed behavioral data. This continuous learning loop would help mitigate model drift, reduce false positives, and maintain robust anomaly detection over time.

#### Self-learning adaptive playbooks

While the current system uses dynamic playbooks, future versions could introduce self-learning playbooks that evolve based on attack patterns. Using reinforcement learning (RL) techniques, the system can learn which response actions yield optimal results in specific attack scenarios. For instance, instead of relying on hard-coded “if-then” logic, playbooks could be enhanced with machine learning to determine the best course of action in real time.

#### Zero trust AI-driven policy engine

An AI-driven policy engine could make Zero Trust decisions more context-aware. Instead of relying on pre-defined rules, the AI engine could evaluate risk in real time using large datasets of user activities. By analyzing contextual inputs like device health, past behavior, and time-of-day patterns, the AI system could apply more sophisticated risk-based policies. This enhancement would reduce the need for static rule-based playbooks.

#### Advanced threat intelligence feeds

The system currently uses threat intelligence to detect malicious IPs during DDoS attacks. Future enhancements could integrate multiple open-source threat intelligence feeds (like VirusTotal, AlienVault OTX) to proactively block threats. The SOAR engine could subscribe to threat feeds, continuously ingest new Indicators of Compromise (IOCs), and update firewall rules automatically.

#### Explainable AI (XAI) for UEBA

One of the main criticisms of machine learning models is their “black-box” nature, particularly in the context of UEBA risk scoring. A potential future enhancement for ZenGuard is the incorporation of Explainable AI (XAI) techniques to make risk evaluation more transparent for users and analysts. Such techniques could highlight the specific user behaviors—such as login time, location, or device type—that contributed to a given risk score, while also providing a clear audit trail of how decisions were reached. In addition, the system could generate human-readable explanations for anomaly alerts, making it easier for security analysts to validate, interpret, and ultimately trust the system’s automated decisions.

## Conclusion

This study proposed ZenGuard, a vendor-neutral framework integrating Zero Trust principles with SIEM, UEBA, and SOAR systems to facilitate continuous user verification, dynamic risk-based access control, and automated incident response. By leveraging behavioral analytics, the framework enabled real-time anomaly detection and adaptive enforcement actions such as MFA challenges and endpoint isolation. Experimental results demonstrated the framework’s effectiveness in mitigating both network and user-level threats, with MTTR under 10 seconds for network attacks (e.g., SYN Flood, DDoS) and 15–25 seconds for user-centric threats such as privilege escalation and session hijacking. Furthermore, the incorporation of Python-driven adaptive playbooks alongside open-source integrations ensured operational flexibility and cost efficiency, positioning ZenGuard as a viable solution for threat detection and response in modern enterprise and hybrid cloud environments.

## Data Availability

It is pertinent to note that all data used was ethically collected from operational SOC environments or safely synthesized, and the dataset is available upon request to encourage reproducibility and further community research.https://github.com/aaminahassan/Zenguard/
